# Experiments and Observations on the Acid Properties of the Yellow Oxyd of Tungsten

**Published:** 1799-05

**Authors:** 


### *Experiments and Obſervations on the acid Properties of the Yellow Oxyd of Tungſten.*

[ *Communicated by a Correſpondent.* ]

WE are indebted, for our firſt accurate ideas of the nature of the metallic ſubſtance called tungſten, to SCHEELE, who affirmed that it conſiſted of calcareous earth, united to a peculiar acid.

His firſt proceſs for obtaining it was, by fuſing one part of tungſten with four of carbonate of potaſs, and diſſolving the melted maſs in boiling water. This water holds in ſolution a ſalt, reſulting from the union of the tungſtic acid with the alkali made uſe of, which is decompoſed on the addition of the ſulphuric, nitric, or muriatic acids, which unite with the potaſs and precipitate the acid of tungſten in the form of a white powder. He afterwards propoſed a ſecond, which conſiſted in digeſting the pulverized tungſten in diluted nitric acid, which diſſolves the calcareous earth, and uncovers the acid of tungſten, which appears in the form of a yellow powder. After repeated edulcorations have freed the powder from any ſenſible portions of acid, it is taken up, by digeſting with cauſtic volatile alkali, which completely diſſolves it. On the addition of an acid, it is precipitated from this ſolution in the form of a white powder, as in the foregoing proceſs, and this powder was, by SCHEELE, and afterwards by BERGMAN, called the pure acid of tungſten.

Wolfram, treated in the ſame manner, afforded alſo a white precipitate, which was ſhewn by Meſſrs. D'ELHUYART, to be the ſame with that obtained by Scheele, from tungſten. They proved likewiſe, by numerous and concluſive experiments, that this white ſubſtance was not the pure acid, but a triple ſalt, reſulting from the union of the tungſtic acid with the potaſs, and the acid employed in the precipitation. The yellow powder uncovered by digeſtion in nitric acid, was, according to the experiments of theſe gentlemen, the pure acid of tungſten.

In the following experiments of VAUQUELIN and HECHT, extracted from the Journal *des Mines*, they have not only confirmed thoſe of D'Elhuyart, but proſecuted their inquiries ſtill further, and aſcertained completely the nature of this metallic acid.

A mixture of three parts of nitrate of potaſs, with one of pulverized wolfram, was thrown into a red-hot crucible, and kept in a ſtate of fuſion for half an hour. The maſs, poured out upon a plate of iron, was of a greeniſh colour, and on cooling, cryſtallized at its ſurface in ſmall needles. It was was dissolved in water, and deposited a brown insoluble matter, which consisted of the oxyds of iron and manganese. The filtered liquor was of a greenish colour, which it was deprived of by boiling, and then again deposited a brownish sediment.

1. On the addition of sulphuric acid to this liquor (which held the tungstate of potash in solution), a white precipitate was formed. The same phenomenon took place on the addition of the nitric, muriatic, acetous, and oxalic acids.

2. On evaporating the liquor resulting from the precipitation of this white matter, a salt was obtained, composed of potash, the acid employed in the precipitation, and the acid of tungsten, which was rendered slightly soluble in water by the potash and acid employed. The solubility of this triple salt was, however, diminished by the further addition of acid.

3. In decomposing the tungstate of potash by the sulphuric acid, there remains in the liquor sulphate of potash, but scarce any acid of tungsten, if a considerable quantity of sulphuric acid has been employed.

4. On adding nitric acid to the solution of tungstate of potash, a salt is obtained from the supernatant liquor, in crystals resembling the scales of fish. This salt contains nitrate of potash*, which had not been decomposed, and a triple salt, composed of the tungstic and nitric acids, and potash; it is soluble in water, and with lime-water forms a white precipitate; it is not affected by the air; it has a sharp, metallic taste; during evaporation, it spreads over the sides of the vessel; when distilled with sulphuric acid, it affords nitric acid, and the retort contains sulphate and tungstate of potash. This residuum, dissolved in water, does not form a yellow powder by ebullition, notwithstanding the acidity of the liquor. It is necessary to separate the sulphate of potash by lixiviation, and boil the white residuum in a concentrated acid. The white salt then assumes a yellow colour, and it is only in this last state, that it is perfectly pure.

5. The acetous acid occasions also a slight precipitate, in the solution of the tungstate of potash, but there is a point at which this precipitate disappears, and does not re-appear, but on the addition of a greater quantity of acetous acid. This salt has at first a taste resembling that of acetite of lead; but it soon after produces a sensation sharp and styptic, resembling that of the metallic salts.

6. The phosphoric acid occasions only a slight precipitate, even when added in considerable quantity. It appears to form a salt extremely soluble.

7. When

* Vauquelin substituted the nitrate of potass for the carbonat in these experiments, which is attended with some advantage in the decomposition of wolfram.

7. When the white ſalt is boiled in concentrated vitriolic acid, diluted with an equal quantity of water, it aſſumes a yellow colour, more or leſs deep; the ſame takes place with the nitric and muriatic acids. The liquor ſeparated from the yellow precipitate affords, by evaporation, a ſalt compoſed of potaſs and the acid employed, with a ſmall quantity of the white ſalt that remained diſſolved.

8. When boiled in acetous acid, it acquires a blue colour, which becomes deeper on evaporation. The liquor when filtered is clear, but depoſits, at the end of ſome days, a white powder, which becomes yellow, on boiling with the ſulphuric acid. The blue powder loſes its fine colour in a great degree by drying.

9. Boiling water is equally capable of diſſolving the white ſalt: the clear liquor depoſits, after ſome time, a white powder.

10. The oxygenated muriatic acid occaſioned no precipitate in the ſolution of tungſtate of potaſs. The white ſalt was not rendered yellow by it; but was diſſolved on ebullition, and again depoſited on cooling, in the form of a white powder. It appears that this ſolution is effected by the water alone in which the oxygenated muriatic gas is diſſolved.

11. Cauſtic potaſs and ſoda, as well as ammoniac, completely diſſolve this triple ſalt. It is an excellent mode of ſeparating the ſilex which always accompanies wolfram.

12. A plate of iron, in contact with the white ſalt, inſtantly produces a fine blue colour. On boiling it with ſmall pieces of iron, and a little water, the colour is equally manifeſted; a ſmall portion of the ſalt is, however, diſſolved, and the reſt preſerves only a greyiſh blue colour.

13. The yellow acid of tungſten, although carefully edulcorated, retains always a ſmall portion of the acid employed in ſeparating it from the potaſs. It may be entirely deprived of it, by ſubjecting it to a red heat in a crucible for ſome time; and it is not till after it has undergone this laſt operation, that it is in a ſtate of perfect purity.

Theſe experiments ſerve to confirm thoſe of D'Elhuyart who had already advanced, that the tungſtic acid of Scheele, or the white powder precipitated by means of an acid, was a triple ſalt, compoſed of potaſs, acid of tungſten, and the acid employed in the proceſs; and that the yellow powder produced by decompoſing tungſten, or wolfram, by means of the nitric or muriatic acid, after the ſecond manner deſcribed by Scheele, was the pure acid of tungſten. From the experiments of Vauquelin, however, it appears, that ſimple edulcoration is not ſufficient to free the yellow powder from any admixture of acid, a ſmall portion of which it obſtinately retains, and on which its acid properties depend. When freed, in the manner before directed, from all remaining portions of acid, it preſented the following properties:—

1. Expoſed to the blow pipe, in a platina ſpoon, it aſſumed a deep green colour: on charcoal it became almoſt black.

2. It diſſolved in borax, without altering either the colour or tranſparency of the globules, even when in conſiderable quantity.—When a very large proportion, however, was employed, it communicated a black, or deep blue colour to it.

3. The phoſphate ammoniacal of ſoda completely diſſolved it, and forms a globule of a deep blue colour.

4. When calcined a long time, with acceſs of air, its yellow colour becomes deeper, and paſſes ſometimes to a green. On heating it during ſeveral hours in a covered crucible, it aſſumed a greyiſh black colour.

5. When thus calcined, it has no taſte. It is not ſoluble in water, and very triflingly ſo in acids. On triturating it with water, it remains ſuſpended for a long time, and forms a kind of yellowiſh emulſion, which does not redden blue vegetable colours. It does not change colour either by expoſure to the ſun or to humidity.

6. It was boiled in concentrated nitric acid, and evaporated to dryneſs: no red vapours were formed. After having repeated this proceſs ſix times ſucceſſively, it was expoſed to a red heat for ſome time, and found to have undergone no change during the operation: it had the ſame colour, and afforded no ſigns of acidity.

It is evident from theſe experiments, that the ſubſtance formed by the combination of tungſten with oxygen, does not poſſeſs thoſe properties which at the preſent day are aſcribed to acids, ſince it is inſoluble in water, does not alter blue vegetable colours, and has no ſenſible taſte. It poſſeſſes, in common with acids, the power of combining with earths, alkalis, and the metals:—but are theſe characters ſufficient to authorize us to place it in the ſame claſs?

From theſe conſiderations it appears neceſſary to exclude this ſubſtance from the number of metallic acids, and rank it amongſt the oxydes; or if we muſt ſtill conſider it as an acid, we ſhall be obliged to regard as ſuch the oxyds of zinc, tin, antimony, and arſenic, which like it unite with alkalis, earths, and ſome other metallic oxyds, with which they form a ſpecies of neutral ſalts. If Scheele, who firſt made known this ſubſtance, has regarded it as an acid, it is becauſe he never obtained it but in a ſtate of triple combination; which exhibits indeed acid properties, becauſe it conſtantly retains a portion of the acid employed in the precipitation, which is proved by the experiments of D'Elhuyart, as well as the foregoing.

This ſubſtance, therefore, known till now under the name of acid of tungſten, ought no longer to be conſidered as ſuch, but as an oxyd of tungſten, and referred to the claſs of metallic oxyds to which it evidently belongs. T.

